# The Associations Among Individual Factors, Media Literacy, and Dietary Supplement Use Among College Students: Cross-Sectional Study

**DOI:** 10.2196/19056

**Published:** 2020-08-31

**Authors:** Shu Ching Yang, Wan-Chen Hsu, Chia-Hsun Chiang

**Affiliations:** 1 National Sun Yat-Sen University Kaohsiung Taiwan; 2 National Kaohsiung University of Science and Technology Kaohsiung Taiwan

**Keywords:** college student, dietary supplement, media literacy, ehealth literacy

## Abstract

**Background:**

The mass media have been condemned for encouraging young people to take dietary supplements (DS). Media literacy, which includes authors and audiences (AA), messages and meanings (MM), and representation and reality (RR) domains, is a new approach to teaching young adults to make better informed health decisions. However, it is not clear which domains are the most important for media literacy education.

**Objective:**

The purpose of this study is to investigate the associations among individual factors, media literacy, and DS use.

**Methods:**

The survey instrument included demographic items, the DS Media Literacy Scale (DSMLS), and DS use items (users or nonusers, types of DS, current use of DS, and intention to use DS in the future). The DSMLS is an 11-item instrument designed to assess college students’ AA, MM, and RR media literacy in relation to DS. A total of 467 Taiwanese college students participated in the study. Descriptive statistical analysis, logistic regression analysis, and multiple regression analysis were conducted.

**Results:**

A total of 338/467 (72.4%) participants reported using DS, and 176/467 (37.7%) consumed 3 or more supplements. Moreover, the MM media literacy domain was associated with having been a DS user (odds ratio 0.63, *P*=.002), current DS use (β=–.10, *P*=.02), and intention to use DS in the future (β=–.12, *P*=.011). Finally, perceived importance of health was positively related to current DS use (β=.18, *P*=.001) and intention to use DS in the future (β=.18, *P*=.001).

**Conclusions:**

This study showed that the majority of Taiwanese college students were DS users and used multiple types of supplements. Moreover, students with lower MM media literacy were more likely to be DS users, to take DS more frequently, and to have higher intentions for future frequent DS use. Finally, those who placed extreme importance on health were more likely to take DS frequently and have higher intentions for future frequent DS use.

## Introduction

In the food industry, the driving force behind the sale of dietary supplements (DSs) is the creation of a market niche to commercialize innovative products claiming to support good health and supplement the diet [1]. DSs are popular in many countries, and their use has been increasing [2]. In the United States, studies have shown that the majority of DS users (79%) reported using DSs every day within the last 30 days [3] and that multivitamin–mineral products were the most common type of DSs [2,4].

Individuals obtain information on DSs most frequently from the media [5]. DSs are accompanied by media messages and advertisements on the need to optimize nutrition and improve health [1]. A proactive, convenient approach to maintaining health is a powerful appeal to consumers [1]. Studies have found that, among DS users and nonusers, the decision to use DSs is influenced by the media [6,7].

Mass media have been condemned for encouraging young people to take DSs [8]. These specific DS products and advertising for DSs are frequently formulated for the young market [2]. A study has shown that compared with the general population, college students are more likely to take DSs and that over 60% of them take 1 or more supplements weekly [2]. Notably, advertising is more likely to increase adolescents’ willingness to use DSs compared with other groups [9]. Individuals are exposed to a large number of diverse media messages, such as health sciences research reports that make sensational claims, in which some specific information is also manipulated for commercial interests [10]. However, adolescents usually do not have the ability to critically analyze the health information received from the media [11]. Scholars have argued that adolescents may take DSs inappropriately due to the questionable information about DSs from magazines [12].

A number of studies have reported that a vast majority of DSs that young people use are not only non-helpful but also often harmful [13]. For example, Or et al. [14] examined the DSs’ harmful effects on children, adolescents, and young adults. Using the Food and Drug Administration adverse event data between 2004 and 2015, they found that, compared with vitamins, teenagers using DSs to lose weight, gain weight, or build muscle are nearly three times more likely to experience serious health problem and even death. Thus, they suggest efforts aimed at reducing access and consumption and actively implementing regulations as well as providing a clear warning and labeling to prevent serious medical consequences for children, adolescents, young people, adults, and general consumers.

Media literacy is a new approach to teaching youth to make smarter health decisions [15]. Media literacy is defined as accessing, understanding, and evaluating messages using different forms of media [16,17]. According to the theory of reasoned action, media literacy programs can potentially reduce the negative influence of media on individuals’ attitudes and normative beliefs [18]. When applied to health, media literacy can help youth understand how products are promoted by the media and increase their awareness about what they see and hear [15]. Media literacy education guides and enables young people to more actively evaluate, analyze, and process mass media messages rather than passively remain targets of the media [19,20]. Such education is designed to empower young people to develop critical thinking abilities to ameliorate the negative effect of mass media messages and make good decisions about their health [21]. Media literacy education has been an effective approach in health behavior areas, such as nutrition behaviors, body image, or alcohol and drugs [22]. Recently, studies have shown that media literacy interventions are effective for decreasing the use of DSs such as creatine, carnitine, and amino acids among adolescents [23] and can reduce young athletes’ use of new sport supplements at the 1-year follow-up [24].

According to Primack et al. [20], media literacy includes 3 domains: “authors and audiences (AA), messages and meanings (MM), and representation and reality (RR)”. Most practitioners and researchers agree that the 3 domains are the foundation of media literacy education [15]. However, it is not clear which domains are the most important for media literacy education [15]. Knowledge of which domains are most important will inform research to design more effective media literacy programs to reduce college students’ DS use. Therefore, this study investigates whether the AA, MM, and RR domains of media literacy were associated with DS use and examines which domain of media literacy exhibits the highest correlation with DS use. We thus propose the following hypotheses:

- H1: College students with lower AA media literacy are more likely to take DSs.
- H2: College students with lower MM media literacy are more likely to take DSs.
- H3: College students with lower RR media literacy are more likely to take DSs.

The study also investigated the influence of demographic characteristics (ie, perceived importance of health, gender, and subjective health status) on DS use. Studies have shown that DS users tend to take DSs to improve or maintain health [2,4,10]. Studies have also found gender differences in DS use. Compared with males, females were more likely to take DSs [1,25,26]. However, male college students were significantly more likely to take at least five types of DSs weekly [2]. In addition, some studies showed that DS users tend to report very good or excellent health [4,25]. However, other studies found an association between poor subjective health and frequent consumption of calcium tablets [1] and DSs [26]. Given the above studies, we want to investigate whether college students’ demographic characteristics (ie, perceived importance of health, gender, and subjective health status) are related to their use of DS.

## Methods

### Study Design and Participants

The participants in this study were college students in Taiwan. After requesting consent from teachers at the selected schools, we distributed the pen-and-paper survey in their classes. We determined that a minimum of 110 students were needed for the pretest (10 times the number of items on the survey instrument) [27], and 350 students were needed for the formal study [28]. The pen-and-paper questionnaires were administered in class during the regularly scheduled class periods. Students took 10-15 minutes to complete the questionnaire and received a small gift as a reward, regardless of whether they completed the survey.

For the pretest, exploratory factor analysis (EFA) was used to assess the reliability of our survey instrument. A sample of 200 college students from 2 schools was recruited to participate in the survey. Ultimately, 73% of the sample returned questionnaires (N=146) and fully completed all the questions in the survey, whereas the other 27% (N=54) did not return the survey. The low rate of returned questionnaires was due to the fact that it was the end of the semester, and thus, some teachers and students were not available.

In the formal study, a sample of 500 college students was drawn from 6 colleges and each member of the sample received a questionnaire. Of the 475 surveys received, 8 surveys were invalid (ie, respondents did not complete the entire survey), whereas 467 were valid. Of the 467 participants, 220/467 (47.1%) were female, and 247/467 (52.9%) were male. The participants’ mean (SD) age was 20.39 (1.88) years.

### Survey Instrument

The questionnaire was composed of items on participants’ demographic information, the DS Media Literacy Scale (DSMLS), and items on DS use.

The demographic information included items on age, gender (male or female), perceived importance of health (1 item with a 5-point Likert scale), and subjective health status (1 item with a 5-point Likert scale).

We developed the DSMLS based on a thorough review of the literature [20,29,30], and we asked 3 specialist professors to examine the content validity of the DSMLS. The DSMLS contains 3 domains (11 items) to measure DS media literacy (Table 1). The AA (4 items) domain evaluates individuals’ skills used to understand how media target specific consumers. The MM domain (3 items) evaluates individuals’ skills used to analyze how messages are created with specific production techniques designed to influence consumers’ attitudes and behaviors. The RR (4 items) domain assesses individuals’ skills used to analyze how media omit and filter information. The items include a 5-point Likert response scale ranging from *strongly disagree* (coded as 1) to *strongly agree* (coded as 5). Higher scores on the respective levels indicate higher media literacy.

The results of the EFA (principal axis factors method) indicate that the Kaiser–Meyer–Olkin measure was 0.84, the Bartlett test for sphericity was significant (*P*<.001), the factor loadings ranged from 0.78 to 0.94 (Table 1), and the explained variance was 78.42%. The DSMLS also exhibited good internal consistency reliability (AA Cronbach α=.94, MM Cronbach α=.79, RR Cronbach α=.89).

**Table 1:** Factor loadings for the DSMLS^a^.

Items	AA^b^ domain	MM^c^ domain	RR^d^ domain
1. Media choose stories of dietary supplements based on what will attract the largest audience	0.94	–0.08	0.25
2. The owners of dietary supplement companies influence the advertising content that is produced	0.85	–0.15	0.31
3. Dietary supplement companies only care about making money	0.78	0.04	0.38
4. Some dietary supplement advertisements are designed to appeal to certain people	0.93	–0.08	0.25
5. Dietary supplement ads convince me that dietary supplements make me healthier and more energetic	–0.06	0.85	–0.03
6. Plots about dietary supplements in TV and movies affect my view of dietary supplements	–0.03	0.88	–0.07
7. I am influenced by celebrity endorsements of dietary supplements	–0.09	0.79	0.02
8. There are often hidden harmful messages in dietary supplement advertisements	0.21	–0.03	0.83
9. Advertisements usually highlight and emphasize the benefits of dietary supplements	0.26	0.06	0.83
10. Advertisements usually leave out some important information regarding dietary supplements	0.37	–0.10	0.80
11. When I see a dietary supplement advertisement, it is very important to think about what was left out of the advertisement.	0.25	–0.06	0.82
Explained variance, %	30.88	19.66	27.88

^a^DSMLS: DS Media Literacy Scale.

^b^AA: authors and audiences.

^c^MM: messages and meanings.

^d^RR: representation and reality.

The survey also included detailed questions on the types of DSs participants used and 2 questions about the frequency of DS use. Participants were instructed to list DSs that they used. The 2 questions about the frequency of DS use (rated on a 5-point Likert scale) included current DS use, defined as the frequency of DS use in the past year, and intention for future DS use, defined as the expected frequency of DS use in the next year.

### Data Analysis

First, we used EFA to assess the reliability of the DSMLS in the pretest study. In the formal study, we used descriptive statistical analysis, logistic regression analysis, and multiple regression analysis. In the regression analyses, gender was a dummy variable (male=0, female=1), and perceived importance of health and subjective health status were continuous variables. The Omnibus test (*P*<.05) was used in the logistic regression to estimate the goodness-of-fit of the model. The variance inflation factor (VIF) and tolerance assessment were used for diagnosing collinearity in multiple regressions. Values of VIF exceeding 4 or values of tolerance less than 0.2 indicate multicollinearity.

## Results

### Descriptive Statistical Analysis

Table 2 presents the descriptive statistics for the categorical variables. Among all participants, the mean (SD) score was 3.35 (0.81) for subjective health status, 3.51 (0.77) for perceived importance of health, 3.56 (0.63) for the AA domain, 3.43 (0.75) for the MM domain, and 3.87 (0.72) for the RR domain, indicating that the college students reported moderate to good health, considered health to be moderately important, and had moderate levels of AA, MM, and RR media literacy, respectively.

Regarding the frequency of DS use, the mean (SD) score for current DS use was 2.27 (1.05), whereas that of intention for future DS use was 2.28 (1.01). This result indicates that college students had occasionally used DSs in the past year and intended to occasionally use DSs in the next year.

Table 3 shows that 338/467 (72.4%) participants reported taking DSs for 1 year before the survey. Overall, 123/467 (26.3%) participants reported taking at least four types of DSs. The most popular DSs were vitamin B (252/467, 54.0%), multivitamins (191/467, 40.9%), and calcium (119/467, 25.5%).

**Table 2:** Descriptive statistics for the categorical variables.

Variables	Mean (SD)
Subjective health status	3.35 (0.81)
Perceived importance of health	3.51 (0.77)
AA^a^ domain of media literacy	3.56 (0.63)
MM^b^ domain of media literacy	3.43 (0.75)
RR^c^ domain of media literacy	3.87 (0.72)
Current DS^d^ use	2.27 (1.05)
Intention for future DS use	2.28 (1.01)

^a^AA: authors and audiences.

^b^MM: messages and meanings.

^c^RR: representation and reality.

^d^DS: dietary supplement.

**Table 3:** Participants’ reported use of DSs (N=467)^a^.

Variable and group		n (%)	
**DS**			
	Nonuser		129 (27.6)
	User		338 (72.4)
**Number of DSs**			
	1		103 (22.1)
	2		59 (12.6)
	3		53 (11.3)
	4+		123 (26.3)
**Top 5 DSs**			
	Vitamin B (B complex)		252 (54.0)
	Multivitamin		191 (40.9)
	Calcium		119 (25.5)
	Iron or zinc		116 (24.8)
	Lutein		43 (9.2)

^a^DS: dietary supplement.

### Analysis of Demographic Characteristics, Media Literacy, and DS Use

In the logistic regression analysis, the Omnibus test (chi-square=17.88, *P*=.007) reveals the goodness-of-fit of the model. In the multiple regression analysis, the values of the VIF range from 0.59 to 0.99, and the values of tolerance range from 1.01 to 1.70, indicating that the data are free from multicollinearity.

The results of the logistic regression analysis and multiple regression analysis are presented in Tables 4 and 5 and show that the MM domain of media literacy was associated with being a DS user (odds ratio 0.63; 95% CI 0.47-0.84; *P*=.002). The MM domain of media literacy was negatively related to current DS use (β=–.10, *P*=.02) and intention for future DS use (β=–.12, *P*=.011). However, the AA and RR domains of media literacy were not related to being a DS user, current DS use, or intention for future DS use. Thus, Hypothesis 2 was supported; however, Hypothesis 1 and 3 were not supported.

Table 5 shows that perceived importance of health was positively related to current DS use (β=.18, *P*=.001) and intention for future DS use (β=.18, *P*=.001). However, Table 4 shows that the association between the perceived importance of health and being a DS user was not statistically significant. Gender and subjective health status were not related to being a DS user, current DS use, or intention for future DS use.

**Table 4:** Logistic regression analysis of DS^a^ use.

Characteristic		DS user		
		Adjusted odds ratio (95% CI)		*P* value
**Gender**				
	Male	1.00^e^		
	Female	0.84 (0.55-1.30)		.47
Subjective health status		1.02 (0.76-1.38)		.90
Perceived importance of health		1.28 (0.94-1.74)		.12
AA^b^ domain of media literacy		0.85 (0.56-1.31)		.46
MM^c^ domain of media literacy		0.63 (0.47-0.84)		.002
RR^d^ domain of media literacy		0.89 (0.61-1.29)		.53

^a^DS: dietary supplement.

^b^AA: authors and audiences.

^c^MM: messages and meanings.

^d^RR: representation and reality.

^e^Male group was used as the reference group. Thus, CI and *P* values were not reported.

**Table 5:** Multiple regression analysis of the frequency of DS^a^ use.

Variable	Current DS use				Intention for future DS use		
	B	β	*P* value	B		β	*P* value
Gender (female)	–0.01	–.00	.93	0.02		.01	.82
Subjective health status	–0.13	–.10	.07	–0.12		–.10	.07
Perceived importance of health	0.24	.18	.001	0.24		.18	.001
AA^b^ domain of media literacy	–0.12	–.07	.24	–0.18		–.11	.06
MM^c^ domain of media literacy	–0.15	–.10	.02	–0.16		–.12	.011
RR^d^ domain of media literacy	0.03	.02	.74	0.04		.03	.66
Model summary	*R*=0.20; *R*^2^=0.04; *F*_6,460_=3.05				*R*=0.22; *R*^2^=0.05; *F*_6,460_=3.87		

^a^DS: dietary supplement.

^b^AA: authors and audiences.

^c^MM: messages and meanings.

^d^RR: representation and reality.

## Discussion

### Principal Findings

This study aimed to investigate the associations between media literacy and DS use in the college-age population. The study revealed that the majority of college students were DS users and used multiple types of supplements. Furthermore, the study found that the MM domain of media literacy showed the highest correlation with DS use. Finally, perceived importance of health was positively related to current DS use and the intention for future DS use.

The study found that 338/467 (72.4%) participants reported using DSs and that 176/467 (37.7%) consumed 3 or more supplements. Among the participants, 252/467 (54.0%) took vitamin B, 191/467 (40.9%) took multivitamins, and 119/467 (25.5%) took calcium. However, few young, healthy students did not intake the required nutrient components [2]. Scholars have recommended that well-nourished adults should stop wasting money on mineral and vitamin supplements; there is no evidence to suggest that these supplements provide any advantage for well-nourished individuals and might even be harmful [31]. Therefore, it is worrying that healthy Taiwanese college students might be more likely to use DSs excessively and thereby harm their health.

As expected, participants with a lower MM media literacy were more likely to be DS users. Moreover, they were more likely to take DSs more frequently and to have higher intentions for future frequent DS use. The MM domain refers to the fact that messages are created with appealing techniques designed to influence consumers’ attitudes and behaviors [20,29,30]. Mass media producers often use certain techniques to make DSs seem healthier and to promote the idea that they *optimize* nutrition. A convenient approach to maintain or improve health is an attractive strategy for individuals [1]. The MM domain of media literacy addresses these miscommunications by exposing these techniques that are designed to make behaviors seem more normative [30]. Students with adequate MM media literacy were less likely to be influenced by media messages and techniques, as they could critically evaluate the messages of DS; thus, they were more likely to be DS nonusers, to take DSs less frequently, and to have lower intentions for future frequent DS use. Therefore, increasing college students’ awareness about how media messages are created to influence their attitudes and behaviors to reduce their use of DSs is a good strategy.

The study found that the AA and RR domains of media literacy were not related to DS users, their current use, or their intention for future use, thus suggesting that MM may be more important than the other 2 domains in terms of their potential relationship with DS-related behavior. These findings suggest that media literacy programs that merely portray the DS industry as manipulative of particular target markets and describe the difference between narrative media and the true effects of DS use on health may not be enough to reduce college students’ DS use. However, it is unlikely that the insufficiency of such a strategy is because the AA and MM concepts are not important. Media literacy is a multifaceted construct, wherein each of the 3 components plays an important role in media literacy and reveals substantial differences in emphasis [20,30]. The AA domain is associated with how profit-driven media target specific market issues, whereas the RR domain focuses on how media information affects one’s perceptions of reality.

Lucidi et al.’s study [23] found that media literacy intervention, which contains a series of interlocking cognitive processes, for example, the acquisition of knowledge structures, diverse cognitive skills, and the minimization of possible detrimental effects of the media, is effective in reducing the use of DSs (ie, creatine, carnitine, and amino acids) among adolescents. Thus, it is important to first discuss who the author of a message is (ie, the AA domain) that erases the distinction between representation and reality (ie, the RR domain) before diving into how media messages are created to affect their attitudes and behaviors (ie, the MM domain). Accordingly, a successful media literacy program relies on 3 interlocking domains (ie, the AA, MM, and RR domains). Of these, as the MM domain is the most strongly associated with DS use, it should probably be the most emphasized. In addition, young people are the most active internet users in today’s world, and because the internet offers a wide variety of information regarding DSs, future studies should consider the potential influence of electronic health (eHealth) literacy and social media appeals versus advertisements on DS use among young people.

Moreover, the study showed that gender and subjective health status were not related to being a DS user, current use, or intention for future DS use. The study sample was college-age students with high access to resources. Studies have shown that individuals with higher education are more likely to use DSs than those with lower education [1,9]. Moreover, college students are among the healthiest populations [2]. The sample homogeneity may have attenuated the effect of subjective health status and gender on DS use. The associations among gender, age, education, subjective health status, and DS use are worth examining in further studies.

Finally, the study found that the perceived importance of health was not related to being a DS user; however, participants who placed the utmost importance on health were more likely to take DSs frequently and to have higher intentions for future frequent DS use. A total of 338/467 (72.4%) participants in the study were DS users. The high percentage of the population using DSs might buffer the effect of the perceived importance of health on DS users. Messages about and advertisements for DSs are attractive because of the potential for individuals to improve and maintain their health [1]. Previous studies have shown that DS users tend to take DSs to improve or maintain health [2,4,10]. Thus, Taiwanese college students who place the utmost importance on health would likely be taking DSs continuously to improve and maintain their health.

### Study Limitations

This study had several limitations. For example, our main media literacy scale is a new scale that has not yet had extensive testing. However, it was based on an established model of media literacy [20]. The study found that the MM domain of media literacy was negatively related to DS use. Future studies should continue to use the same framework or other models to develop and construct other DSMLS instruments. Second, the study found that vitamin B, multivitamins, and calcium were the popular types of supplements among Taiwanese college students. Future studies should continue to analyze the associations among the 3 domains of media literacy and the frequency of each type of DS use. Third, many detailed demographic characteristics (ie, body mass index, yearly income, diet category, parental attitudes toward DSs, and parental DS use) were not collected as part of this study. Thus, a broad range of factors that might be influential in determining college students’ media literacy and DS use should be taken into consideration. Finally, this was a cross-sectional study. The intention for future DS use cannot be taken as actual DS use in the next year. Future investigations should examine the associations between media literacy and DS use longitudinally. The study, however, provides a crucial starting point for such studies.

### Conclusions

Mass media have been condemned for encouraging young people to take DSs [8], and DS media literacy is an important subset of media literacy that requires independent investigation. To the best of our knowledge, this study is the first to provide evidence for the associations between DS media literacy and DS use in the college-age population. This study revealed that the majority of the college students were DS users and used multiple types of supplements. Thus, discouraging potentially unnecessary DS use is an important consideration in college health education programs.

The study found that the MM domain of media literacy had the highest correlation with low DS use. This finding suggests that to be most effective, media literacy programs designed for college students should more closely examine the various manipulative marketing techniques of media.

Furthermore, for media literacy programs to be successful, future DS-related media literacy using Primack et al.’s theoretical models can be well designed and integrated via school-based experimental interventions. It is suggested to examine the relationship between DS use outcomes and items measuring specific, individual components of media literacy, and to further examine how well the program buffers the impact of mass media on adolescents’ DS use by empowering youth to actively and critically analyze and evaluate these media messages rather than being passive targets of the messages. Ultimately, this study found that participants who placed the utmost importance on health were more likely to take DSs frequently and have higher intentions for future DS use. Therefore, future studies should pay more attention to college students who place the utmost importance on health, thereby helping them make healthy choices and choose appropriate DSs according to their health situations.

## Abbreviations

AA: authors and audiences
DS: dietary supplement
DSMLS: DS Media Literacy Scale
EFA: exploratory factor analysis
MM: messages and meanings
RR: representation and reality
VIF: variance inflation factor
